# HyKey: hyperspectral keypoint detection and matching in minimally invasive surgery

**DOI:** 10.1007/s11548-026-03633-z

**Published:** 2026-04-27

**Authors:** Alexander Saikia, Chiara Di Vece, Zhehua Mao, Sierra Bonilla, Chloe He, Joao Ramalhinho, Tobias Czempiel, Sophia Bano, Danail Stoyanov

**Affiliations:** 1 Department of Medical Physics and Biomedical Engineering, UCL Hawkes Institute, University College London, London, WC1E 6BT UK; 2 Department of Computer Science, University College London, London, WC1E 6BT UK; 3 EnAcuity Ltd., London, EC2A 4NE UK

**Keywords:** Hyperspectral imaging, Keypoint detection and description, 3D reconstruction, Minimally invasive surgery

## Abstract

**Purpose:**

3D reconstruction in minimally invasive surgery (MIS) enables enhanced surgical guidance through improved visualisation, tool tracking, and augmented reality. However, traditional RGB-based keypoint detection and matching pipelines struggle with surgical challenges, such as poor texture and complex illumination. We investigate whether using snapshot hyperspectral imaging (HSI) can provide improved results on keypoint detection and matching surgical scenes.

**Methods:**

We developed HyKey, a hyperspectral keypoint detection and description model made up of a hybrid 3D-2D convolutional neural network that jointly extracts spatial-spectral features from HSI. The model was trained using synthetic homographic augmentation and epipolar geometry constraints on a robotically acquired dual-camera RGB-HSI laparoscopic dataset of ex vivo organs with calibrated camera poses. We benchmarked performance against established RGB-based methods, including SuperPoint and ALIKE.

**Results:**

Our HSI-based model outperformed RGB baselines on registered RGB frames, achieving 96.62% mean matching accuracy and 67.18% mean average accuracy at 10$^\circ$ on pose estimation, demonstrating consistent improvements across multiple evaluation metrics.

**Conclusion:**

Integrating spectral information from an HSI cube offers a promising approach for robust monocular 3D reconstruction in MIS, addressing limitations of texture-poor surgical environments through enhanced spectral-spatial feature discrimination. Our model and dataset are available at https://github.com/alexsaikia/HyKey-Hyperspectral-Keypoint-Detection.

## Introduction

Hyperspectral imaging (HSI) is an emerging modality with strong potential for intraoperative use [1–3]. By combining imaging and spectroscopy, HSI enables the extraction of both spatial and biochemical information, such as tissue composition, haemoglobin saturation, and morphology, supporting identification of pathological changes, tissue segmentation, and guidance during interventions where subtle spectral differences are invisible to both human vision and standard red-green-blue (RGB) cameras. Traditional RGB endoscopic imaging does not provide sufficient biochemical or morphological information to support these advanced surgical tasks. Moreover, HSI alone is not sufficient in the operating room, as surgeons need both geometry and physiology. If we can reconstruct the operative field in 3D and attach spectral information per surface patch, we can visualise perfusion heterogeneity around anastomoses, support margin assessment, and stabilise augmented reality (AR) overlays, providing capabilities beyond standard RGB endoscopy.

While 3D reconstruction techniques like structure-from-motion (SfM) [4, 5] and simultaneous localisation and mapping (SLAM) [6] have been combined with deep learning (DL) to improve accuracy and robustness in RGB computer vision tasks, adapting these methods to minimally invasive surgery (MIS) challenges, such as tissue deformation, specular highlights, poor texture, occlusions, and varying illumination, remains difficult. As a result, keypoints detection becomes unstable and descriptors drift, which degrades matching and downstream SfM/SLAM. Reliable ground truth for evaluation, such as absolute camera poses or 3D point clouds, is also challenging to obtain. Combining 3D reconstruction with spectral data offers the possibility of enriched surgical maps that visualise anatomy in 3D while providing biochemical context to guide surgical decisions.

**Fig. 1:**
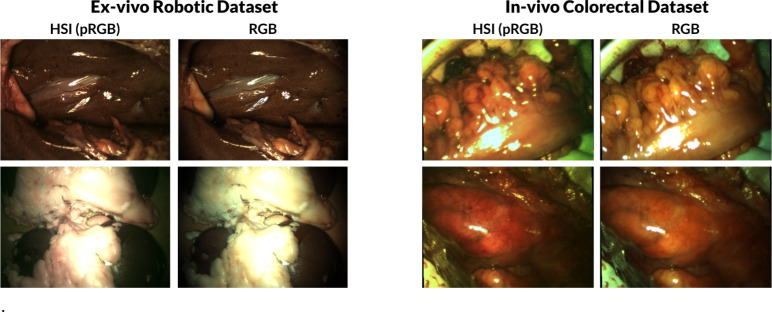
Example frames from the proposed dual-modality datasets. Each includes co-registered hyperspectral (visualised as pRGB) and RGB images. The close spatial alignment between modalities ensures reliable evaluation between RGB baselines and HSI models

Keypoint detection and description are a fundamental step in 3D reconstruction, with classical hand-crafted methods such as scale-invariant feature transform (SIFT) [7], speeded-up robust features (SURF) [8], and Oriented FAST and Rotated BRIEF (ORB) [9] forming the basis for matching and SfM in traditional RGB imaging. While these methods offer scale invariance and efficiency, they remain sensitive to viewpoint changes, non-rigid deformation, and challenging photometric conditions common in surgical scenes, leading to unstable keypoints and degraded matching. With advances in DL, more sophisticated detectors have emerged, including SuperPoint (SP) [10], Accurate and Lightweight Keypoint Detection and Descriptor Extraction (ALIKE) [11], and A LIghter Keypoint and Descriptor Extraction with Deformable transformation (ALIKED) [12], which have improved spatial localisation by learning more robust features from large annotated or synthetic datasets. However, these RGB-based detectors are fundamentally built on 2D convolutions and are limited in their ability to process the full spectral content of an HSI cube. The only prior work extending learning-based detection and description to hyperspectral data, HyperSpectral SuperPoint Network (HSSPN) [13], remains restricted to 2D convolutional architectures, treating each spectral band as an independent channel. While 2D CNNs mix channels through learned weights, stacking bands as channels treats wavelengths like a set of generic channels rather than an explicit measurement axis. Since an HSI cube is naturally represented as a 3D volume, prior work has argued that 3D filtering provides a simple way to jointly extract spectral-spatial features jointly and hybrid 3D-2D designs use early 3D layers to form joint representations before switching to 2D processing for efficiency [14, 15].

To overcome these limitations, we propose HyKey, a spectral-spatial detector and descriptor for HSI in MIS with a 3D-2D backbone and differentiable keypoint detection, trained via homography and epipolar supervision on a dual-modality RGB-HSI dataset (robotic ex vivo and in vivo videos). Our main contributions are as follows:

- **Spectral-spatial features for HSI:** We design a compact 3D-2D CNN that learns inter-band spectral-spatial cues and outputs a score map, keypoints and dense descriptors with a differentiable keypoint detection head for end-to-end training.
- **Geometry-aware supervision:** Our training couples synthetic homography objectives with an epipolar regulariser to minimise Sampson distances without loss of differentiability, leading to improved relative pose recovery.
- **Dual-modality laparoscopic dataset**: We acquire two dual-modality (HSI + registered RGB) datasets in robotic ex vivo laparoscopic organ imagery (released with this work) and in vivo colorectal procedures, enabling benchmarking of HSI matching vs RGB baselines.
- **Comprehensive benchmarking:** We report a detailed evaluation showing that HyKey improves homography metrics and relative pose over RGB and HSI baselines.

## Methodology

### Dual-modality dataset description

To train, validate, and benchmark our model and baselines, we utilise two in-house datasets acquired using a custom dual-modality laparoscope (DML) as illustrated in Fig. 1.

***Dual-Modality Laparoscope*** We acquired two datasets with the same custom-built DML, equipped with both an HSI and an RGB camera. The HSI camera (MQ022HG-IM-SM4x4, XIMEA GmbH, Muenster, Germany) is a $4~\times ~4$ mosaic snapshot camera capturing 16 spectral bands from 460 to 600 nm and a resolution after demosaicing to $272~\times ~512~\times ~16$. The RGB camera is a FLIR Blackfly S USB3 (BFS-U3-50S5C-C, Teledyne FLIR, Wilsonville, Oregon, USA), with a raw resolution of $2448 ~\times ~ 2048$. Both cameras are attached to a beamsplitter that is custom-mounted onto a HOPKINS^®^ Telescope 26003 AGA (Karl Storz SE & Co. KG, Tuttlingen, Germany), therefore aligning their principal axes. Our in-house acquisition software captures a pair of corresponding RGB and HSI frames at each time point. Due to the different aspect ratios and camera orientations, we register the RGB to the HSI cube using keypoints from a Charuco calibration dataset. This enables fair comparison between both modalities.

***In-house Ex vivo Robotic Dataset*** Our in-house robotic dataset was collected using a camera-agnostic robotic arm platform [16] using a 7 degrees-of-freedom manipulator to position the laparoscope around ex vivo ovine organs. Eye-in-hand calibration was performed using a Charuco board as per the paper. Following [16], we collected synchronised RGB-HSI video sequences under the same multiple lighting and trajectory scenarios. The dataset includes 10 ovine kidneys, with some paired up to form a total of 7 organ sets, and 3 ovine livers, and includes multiple recordings of each organ under different acquisition scenarios.

**Fig. 2:**
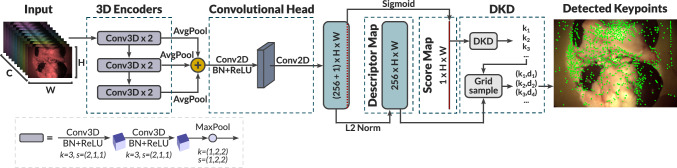
Architecture of the proposed HyKey network for keypoint detection and description in HSI. The network uses three sequential 3D convolutional encoder blocks to extract spatial-spectral features, followed by feature aggregation and upsampling. A 2D convolutional head produces an L2-normalised descriptor map and a keypoint score map, which feeds a DKD module for end-to-end trainable keypoint extraction

All acquisitions were performed using a 0$^\circ$  30 cm 10 mm diameter HOPKINS laparoscope. The different lighting scenarios involved an open surgical light source or the laparoscopic light source. The different trajectory scenarios involved both unconstrained, externally controlled hemispherical sweeps and constrained movements mimicking the remote centre of motion about a trocar port.

In total, the dataset consists of 52 videos, for a total of 41,449 RGB-HSI frame pairs. For evaluation, videos of one kidney and one liver were held out as an unseen test set (9150 frames), and a different kidney–liver pair was reserved as a validation control (5544 frames). While the ex vivo setting differs from in vivo MIS conditions, the dataset effectively captures the spatial-spectral characteristics relevant for keypoint learning, making it well suited for training and evaluation. We are releasing this dataset alongside this work.

***In-house In vivo Colorectal Dataset*** To address the domain gap associated with the robotic ex vivo dataset, we additionally collected an in vivo dataset from 12 patients consented for laparoscopic colorectal cancer resection. The study received ethical approval from the Yorkshire & The Humber - Bradford Leeds Research Ethics Committee (Reference: 24/YH/0088; IRAS project ID: 331933) and was sponsored by Imperial College London. Hyperspectral and RGB images were acquired from multiple views of the tumour region, both in vivo and ex vivo after resection. We removed two patients’ videos for the test set (11,912 frames), a single patient for the validation set (8711 frames) with the training set containing the other nine patients (61,995 frames). Due to patient confidentiality, this dataset is not available for public release.

### Proposed HyKey model

Given an HSI cube $I\in \mathbb {R}^{C,H,W}$ (with $C=16$ bands in our system), HyKey aims at (i) detecting a set of repeatable keypoints and (ii) computing robust local descriptors so that correspondences between views can be established reliably. These correspondences can then feed standard geometric estimators (e.g. homography, essential matrix) to enable 3D reconstruction and camera tracking in MIS scenes.

HyKey adopts a hybrid 3D-2D CNN tailored to HSI (Fig. 2). 3D convolutions capture spectral-spatial correlations early, while a lightweight 2D head produces a keypoint score map and dense descriptors at full image resolution. We prefer this design over commonly reported transformer-heavy models for other HSI tasks [17, 18] because surgical HSI training data are scarce and real-time constraints make large transformers impractical in the operating room. In addition, to the authors’ knowledge, there are currently no transformer-based models focusing on hyperspectral keypoint detection and matching.

#### Architecture details

***3D Spectral-Spatial Encoder*** The HSI cube *I* passes through three 3D convolution blocks, with each block containing two 3D convolutional layers with kernel size $3~\times ~3~\times ~3$ and stride (2, 1, 1), thus halving the spectral bands at each convolution. Each block outputs a different number of channels $c_i$ ($c_1=32$, $c_2=64$, $c_3=128$), with both convolutional layers within each block producing the same number of output channels. After the two convolutions, a spatial max pooling operation (kernel 1, 2, 2) is applied. This produces an output shape of $[c_i,C_{in}/4,H_{in}/2,W_{in}/2]$.

***Feature Aggregation*** The outputs from the three encoder blocks are aggregated using an average pooling across the spectral dimensions, collapsing them to a single channel. After squeezing this singleton dimension, each of the three feature blocks has shape $[c_i,H/2^i,W/2^i]$. These three feature maps are then upsampled to the original spatial resolution $(H_{\text {orig}}, W_{\text {orig}})$ via bilinear interpolation and concatenated along the channel dimensions, producing an aggregated feature block of $F \in \mathbb {R}^{(c_1+c_2+c_3)~\times ~ H_{\text {orig}}~\times ~ W_{\text {orig}}}$.

***2D Convolutional Head*** We apply two $3~\times ~3$ convolutions to *F* to obtain detection scores and descriptors. The first convolution produces *D* channels and is followed by batch normalisation and ReLU. The second convolution produces $D{+}1$ channels, which are split into a score map *S* (sigmoid activation) and a *D*-channel descriptor map; the descriptor vectors are *L*2-normalised per pixel.

***Differentiable Keypoint Detection*** The score map *S* is fed to the differentiable keypoint detection (DKD) module as detailed in [11, 12]. Unlike conventional approaches that apply non-maximum suppression (NMS) directly to the score map, which breaks differentiability and prevents end-to-end training, the DKD module enables differentiable keypoint extraction through soft selection within a local window. This preserves gradient flow throughout the network, allowing joint optimisation of the encoder, decoder, and keypoint detection stages. Descriptors are extracted by grid sampling the descriptor map at the keypoint spatial location.

### Model optimisation

**Fig. 3:**
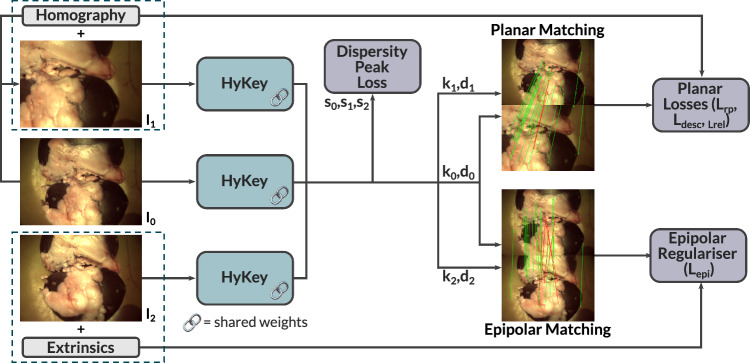
Overview of the HyKey training pipeline. An input frame $I_0$ is paired with a homographically warped version $I_1$ for synthetic supervision. For the robotic dataset, an additional sequential frame $I_2$ from the same video provides a real second view. All inputs are processed by HyKey individually to extract $s_i,k_i,d_i$, score maps, keypoints, and descriptors, which are jointly optimised using planar and epipolar matching constraints

Each training sample consists of three images: a base view $I_0$, a planar homographic view $I_1~=~W_{\textbf{H}_{01}}(I_0)$, and an epipolar view $I_2$ (for robotic data only) with known camera intrinsics *K* and extrinsics $(\textbf{R}_{02}, \textbf{t}_{02})$. For each image, the network predicts keypoints $\mathcal {K}_i$, descriptors $\mathcal {D}_i$, and a score map $\textbf{S}_i$. Descriptor correspondences are established through mutual nearest neighbour (MNN) matching to maintain matcher-agnostic consistency. One-to-one matches are enforced by retaining only mutually consistent correspondences, where keypoint *i* from one image matches keypoint *j* from another image if and only if *j* matches back to *i*; keypoints without such mutual matches are excluded. HyKey is only trained on HSI data under the mixed planar-epipolar supervision regime described below. An overview of the training pipeline is presented in Fig. 3. We combine a planar supervision term from $(I_0,I_1)$ with a geometry-aware term from $(I_0,I_2)$ to get the total loss as:

1 $$\begin{aligned} \mathcal {L}_{\text {total}}= &   \lambda _{\text {pk}}\,\mathcal {L}_{\text {pk}} + \lambda _{\text {rp}}\,\mathcal {L}_{\text {rp}} + \lambda _{\text {rel}}\,\mathcal {L}_{\text {rel}} + \lambda _{\text {desc}}\,\mathcal {L}_{\text {desc}}\nonumber \\  &   \quad +\lambda _{\text {epi}}\,\mathcal {L}_{\text {epi}}. \end{aligned}$$

This joint formulation balances appearance, localisation, and geometric consistency across planar and epipolar pairs. Each loss term is detailed below and is weighted as follows: $\lambda _{\text {pk}}~=~0.5$, $\lambda _{\text {rp}}~=~1$, $\lambda _{\text {rel}}~=~1$, $\lambda _{\text {desc}}~=~5$, and $\lambda _{\text {epi}}~=~0.25$, with the planar loss weights inspired by [12] and the epipolar loss set empirically.

**Dispersity peak loss **$\mathcal {L}_{\text {pk}}$ is applied independently to each image’s score map $\textbf{S}_i$ ($i~\in ~\{0,1\}$), encouraging sharp and localised peaks corresponding to keypoints. It penalises diffuse activations within a $5~\times ~5$ neighbourhood, using a sigmoid-based weighting of the keypoint confidence centred at a score threshold $t_{\text {sc}}$. This term promotes spatially distinct and reliable detections prior to any geometric supervision.

**Planar homography losses **($\mathcal {L}_{\text {rp}}, \mathcal {L}_{\text {desc}},\mathcal {L}_{\text {rel}}$): For the planar image pair $(I_0, I_1)$, we adopt an ALIKE-style self-supervised framework. Keypoints from $I_0$ are projected to $I_1$ via the known homography $\textbf{H}_{01}$ to establish pseudo-ground-truth correspondences, while random locations are additionally sampled to promote descriptor diversity. Three geometric losses are applied: (i) The reprojection localisation loss $\mathcal {L}_{\text {rp}}$ penalises geometric inconsistency between projected and detected keypoints using a Huber loss on the reprojection error, weighted by the product of sigmoid weights from both views; (ii) the sparse score map reliability loss $\mathcal {L}_{\text {rel}}$ encourages confidence consistency between matching keypoints across score maps and descriptor similarities; and (iii) the descriptor reprojection loss $\mathcal {L}_{\text {desc}}$ aligns descriptors across homographic pairs by optimising a cosine-similarity matrix via cross-entropy with symmetric supervision.

**Geometry-aware epipolar loss **$\mathcal {L}_{\text {epi}}$: For the non-planar image pair $(I_0, I_2)$, geometric consistency is enforced through the fundamental matrix $\textbf{F}_{02}~=~\textbf{K}_{2}^{-T}[\textbf{t}_{02}]_\times \textbf{R}_{02}\textbf{K}_{0}^{-1}$, which constrains corresponding keypoints to lie on epipolar lines. The epipolar regulariser $\mathcal {L}_{\text {epi}}$ computes soft correspondence probabilities from descriptor similarities and minimises the expected Sampson error over candidate correspondences using a Huber loss symmetrically in both directions. This encourages robust descriptor learning across non-planar, tissue-like surfaces and complements the planar geometric supervision.

## Experimental setup

For this work, we train two models: HyKey-noPE (no positional epipolar regulariser enabled) and HyKey. For HyKey, it was activated after the first 5 epochs to allow stable descriptor learning. During training, a maximum of 400 detected keypoints with the highest scores and 400 random keypoints were used. The DKD and planar loss hyperparameters followed the configuration from [12].

The models were trained using the Adam optimiser with a learning rate of $3~\times ~10^{-4}$, linearly warmed up over the first 500 steps. A batch size of 6 was used, and to account for varying dataset sizes, training was capped at 10,000 frames per epoch with half of each batch coming from each dataset. All experiments were conducted on a workstation equipped with an AMD Ryzen Threadripper PRO 5975WX CPU and a single NVIDIA RTX A6000 GPU, requiring approximately 43 h for one training run.

### Evaluation metrics

Following previous work [10–12, 19] we utilise the following planar metrics:

- **Repeatability**: proportion of keypoints that have the possibility to be correctly re-detected in the corresponding image within a pixel threshold $\tau$.
- **Matching Score (MS)**: proportion of keypoints successfully matched between image pairs under a pixel threshold $\tau$.
- **Mean Matching Accuracy (MMA)**: proportion of matches with reprojection error below $\tau$, indicating geometric matching accuracy.
- **Mean Homography Accuracy (MHA)**: proportion of image corners correctly aligned within $\tau$ after applying the homography estimated from the predicted matches.

**Table 1:** Loss ablation on the test sets

Model	Loss terms enabled (Eq. (1))					Metrics		
	$\mathcal {L}_{\text {pk}}$	$\mathcal {L}_{\text {rp}}$	$\mathcal {L}_{\text {rel}}$	$\mathcal {L}_{\text {desc}}$	$\mathcal {L}_{\text {epi}}$	Rep AUC	MS AUC	mAA@10$^\circ$
			$\checkmark$	$\checkmark$		64.42	61.87	28.34
	$\checkmark$		$\checkmark$	$\checkmark$		69.17	64.51	52.18
		$\checkmark$	$\checkmark$	$\checkmark$		67.25	65.15	42.90
			$\checkmark$	$\checkmark$	$\checkmark$	67.25	62.78	53.30
	$\checkmark$	$\checkmark$		$\checkmark$		71.53	68.51	44.44
	$\checkmark$	$\checkmark$	$\checkmark$	$\checkmark$		72.47	69.25	51.50
	$\checkmark$		$\checkmark$	$\checkmark$	$\checkmark$	69.20	63.27	65.96
		$\checkmark$	$\checkmark$	$\checkmark$	$\checkmark$	70.22	65.79	58.56
HyKey-noPE	$\checkmark$	$\checkmark$	$\checkmark$	$\checkmark$		**82**.**46**	**80**.**68**	57.48
HyKey	$\checkmark$	$\checkmark$	$\checkmark$	$\checkmark$	$\checkmark$	75.83	72.05	**67**.**18**

Rep/MS are AUC (%) under synthetic homographies; mAA@10$^\circ$ is relative pose accuracy (%) on real video pairs solely from the ex vivo robotic dataset (best bold, second underlined). $\checkmark$ indicates the corresponding loss term in Eq. (1) is enabled

The repeatability and MS metrics are proportional to the total number of keypoints that would be co-visible in each image. For relative pose, we evaluate **mean average accuracy (mAA)**, representing the proportion of predicted poses with angular error below a threshold.

### Model comparison and evaluation settings

We evaluate our HyKey model against both trained/fine-tuned HSI models (HSSPN, HS-ALIKE) and RGB baselines (SIFT, SP, ALIKE) on the in vivo colorectal and ex vivo robotic test sets. For parity across modalities, we register RGB frames to the HSI reference, then crop both to remove invalid regions. The registration direction was chosen so as to not introduce a confounding advantage towards the higher-resolution RGB camera. For evaluation, the model and baselines were set to return a maximum number of 1024 keypoints. We evaluate without test-time augmentation and use MNN matching to ensure fair comparison, avoiding additional supervision from learned neural matchers. We report two complementary settings:

1. Synthetic homography tests on both test sets with Rep, MS, MMA, MHA at thresholds of $\tau ~=~[1,3,5,10,20]$ and evaluate the area under the curve (AUC).
2. Real video pairs (ex vivo robotic test set with ground-truth poses) with mAA@5$^\circ$,10$^\circ$,20$^\circ$ using robust estimator USAC-MAGSAC [20] (threshold = 1 normalised px, confidence = 0.99999) to obtain the fundamental matrix.

## Results and discussion

### Loss function ablation

We ablate the loss components in Eq. (1) to quantify their effects on planar localisation/matching and non-planar geometric consistency. We evaluate (i) Repeatability-AUC and MS AUC under synthetic homography warps on both datasets and (ii) relative pose accuracy (mAA@10$^\circ$) on real video pairs from the ex vivo robotic dataset.

Table 1 shows two main effects. First, using only the core matching terms $\mathcal {L}_{\text {rel}}{+}\mathcal {L}_{\text {desc}}$ yields the lowest performance, indicating that descriptor agreement and score reliability alone are insufficient for stable localisation and matching. Adding localisation supervision improves planar performance, with further gains when $\mathcal {L}_{\text {pk}}$ and $\mathcal {L}_{\text {rp}}$ are combined.

Second, $\mathcal {L}_{\text {epi}}$ primarily improves pose accuracy on real video pairs. Across the ablations, epipolar supervision consistently improves relative pose accuracy. This trend is reflected in the full HyKey model, where adding $\mathcal {L}_{\text {epi}}$ improves pose accuracy over HyKey-noPE. Conversely, HyKey-noPE attains the best planar AUC, indicating a trade-off between planar alignment and improved downstream 3D motion estimation.

### Synthetic homography evaluation

**Table 2:** Homography evaluation on in vivo and ex vivo test sets

	Modality	Rep AUC	MS AUC	MMA AUC	MHA AUC
*In vivo colorectal dataset*					
SIFT [7]	RGB	62.26	51.22	80.42	98.05
SuperPoint [10]	RGB	58.80	25.60	62.03	47.03
RGB-ALIKE [11]	RGB	68.05	61.35	95.03	97.42
HS-ALIKE	HSI	64.56	57.21	93.66	98.52
HSSPN [13]	HSI	66.26	45.54	76.90	79.29
HyKey-noPE	HSI	**79**.**17**	**77**.**67**	**97**.**96**	**99**.**45**
HyKey	HSI	71.15	67.73	95.42	98.18
*Ex vivo Robotic dataset*					
SIFT [7]	RGB	65.77	55.66	83.25	98.62
SuperPoint [10]	RGB	63.28	32.67	69.87	61.86
RGB-ALIKE [11]	RGB	70.93	64.05	95.11	98.37
HS-ALIKE	HSI	74.08	67.21	96.78	99.10
HSSPN [13]	HSI	71.94	53.19	83.56	83.83
HyKey-noPE	HSI	**82**.**46**	**80**.**68**	**98**.**31**	**99**.**58**
HyKey	HSI	75.83	72.05	96.62	98.52

We report Rep/MS/MMA/MHA as AUC (best bold, second underlined). HyKey variants use HSI; RGB baselines use registered RGB; HS-ALIKE/HSSPN use HSI. “noPE” = ablation without positional/epipolar term. Bold = ours

Table 2 summarises homography metrics on the in vivo and ex vivo datasets. HyKey-noPE achieves the highest scores across the homography metrics, while HyKey remains competitive but slightly lower. On the in vivo set, HyKey-noPE outperforms both RGB and HSI baselines across all homography metrics. A similar trend is observed on the ex vivo set, where HyKey-noPE again achieves the best performance and HyKey remains competitive. Both HyKey variants outperform the RGB baselines across all evaluated metrics. These gains reflect improved match density (MS) and higher geometric precision (MMA) in surgical scenes where RGB texture is weak.

**Fig. 4:**
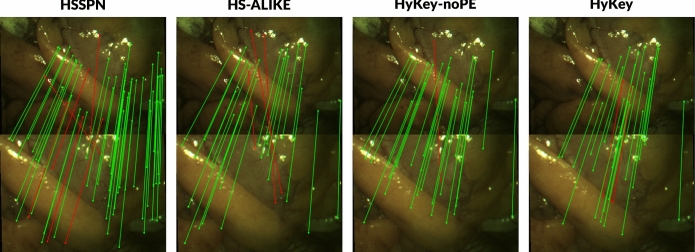
HSI model performance on synthetic homography warping on the in vivo colorectal dataset. The HyKey models have fewer errors, despite the lack of texture in the scene. Green matches dictate a correct match under three pixels. The number of good and bad matches shown has been proportionally downsampled for visualisation

**Table 3:** Relative pose accuracy (mAA) on video sequence pairs using essential matrix recovery

Model	Modality	mAA@5 (%)	mAA@10 (%)	mAA@20 (%)
SIFT [7]	RGB	21.82	35.70	45.90
SuperPoint [10]	RGB	22.60	49.98	76.68
RGB-ALIKE [11]	RGB	32.56	61.56	87.70
HS-ALIKE	HSI	37.76	63.14	87.14
HSSPN [13]	HSI	32.84	59.20	83.32
HyKey-noPE	HSI	32.34	57.48	79.10
HyKey	HSI	**38**.**44**	**67**.**18**	**88**.**74**

We report mAA@5, mAA@10, mAA@20 (best bold, second underlined). HyKey variants and HS-ALIKE/HSSPN use HSI; RGB baselines use registered RGB. “noPE” = ablation without positional/epipolar term. Bold = ours

This evaluation uses synthetic homography augmentation with known planar warps and photometric variation from differing lighting conditions to assess feature localisation and descriptor accuracy under geometric and illumination changes. Modelling joint spectral-spatial cues with 3D-2D convolutions yields stable descriptors under these transformations, while the slightly higher performance of the noPE variant suggests that epipolar regularisation is less beneficial for purely planar motion. Both HyKey variants demonstrate that hyperspectral features remain discriminative even in visually homogeneous tissue, where RGB-based keypoints often fail.

**Fig. 5:**
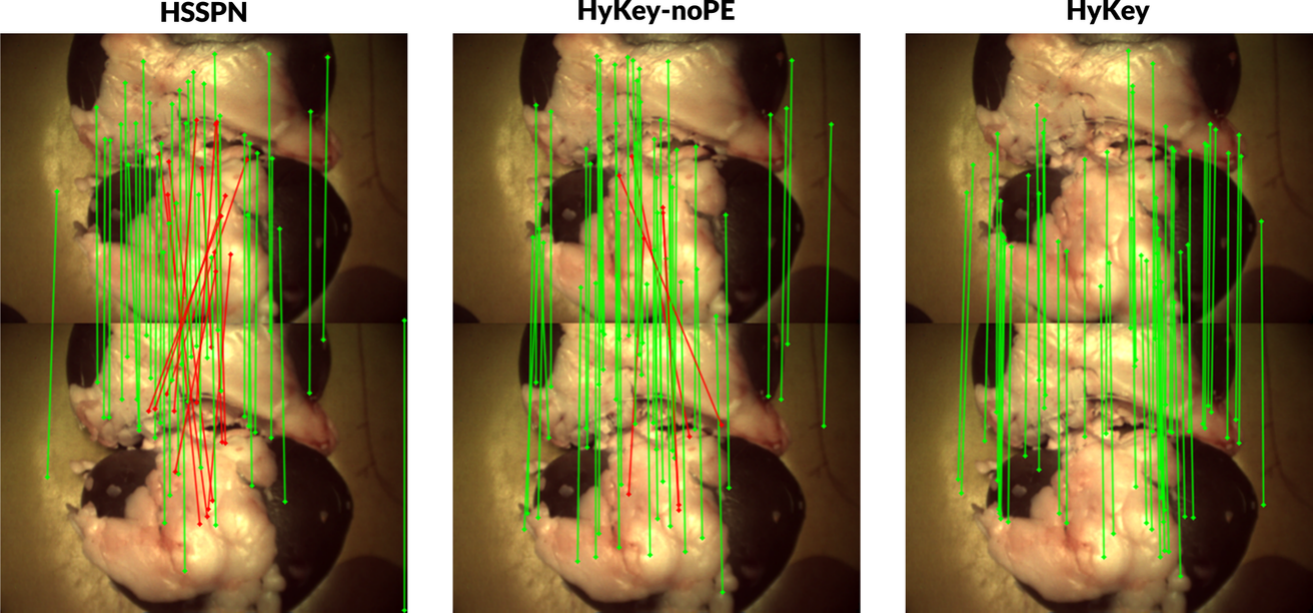
Qualitative view of our model performance on a kidney scene from the robotic ex vivo test set against HSSPN [13], the only learning-based model for keypoint detection on a pair of video frames. A green match indicates the match has an epipolar error less than 5 pixels. Number of keypoints downsampled for visualisation

Figure 4 illustrates the difficulty of these planar scenes, where specular glare and limited texture make correspondences ambiguous even to the human eye. HSSPN and HS-ALIKE frequently misalign in specular regions, whereas both HyKey variants maintain stable and accurate matches across these challenging areas.

The HyKey model runs with an average inference frames-per-second (fps) of 23.53 Hz, showing the real-time capabilities of the model. With future optimisation, this could be incorporated into a real-time SfM pipeline.

### Real video pairs: relative pose evaluation

Table 3 reports model performance on real, non-planar video pairs via relative pose estimation. Unlike the synthetic homography tests, where ground-truth point correspondences are known from the applied warp, true sequences do not provide GT correspondences. However, our robotic platform provides ground-truth camera poses, so we assess correspondence quality by relative pose accuracy (mAA@5$^\circ$, 10$^\circ$,20$^\circ$).

HyKey achieves mAA@10$^\circ$ = 67.18% and mAA@20$^\circ$ = 88.74%, improving over HS-ALIKE (63.14%, 87.14%) and SuperPoint (49.98%, 76.68%). HyKey-noPE is weaker on pose metrics (57.48%, 79.10%), consistent with the intuition that explicit epipolar supervision helps on real non-planar motion, even if pure homography metrics slightly favour our noPE variant. Figure 5 shows the matching performance on the robotic-assisted kidney scene in the test set, where both versions of HyKey outperform HSSPN with fewer erroneous matches.

The largest improvement of HyKey over its noPE variant appears in relative pose accuracy (mAA), confirming that epipolar supervision strengthens geometric consistency beyond planar alignment. Compared to RGB models, hyperspectral methods perform best at smaller angular thresholds (mAA@10$^\circ$), suggesting that the model learns to match tissue regions that are spatially similar but differ subtly in their spectral fingerprints-reflectance patterns invisible to RGB. Such spectral sensitivity provides a clear advantage for fine-grained correspondence in anatomically homogeneous areas that appear visually uniform in RGB.

Figure 5 shows qualitative results on real laparoscopic pairs from the relative pose evaluation. HSSPN and HyKey-noPE produce locally correct but directionally inconsistent matches, while the full HyKey variant preserves coherent correspondence directions consistent with the epipolar geometry.

### Hyperspectral performance against RGB

To compare the effect of imaging modality, we compare hyperspectral models (HS-ALIKE, HSSPN) against their closest RGB counterparts (RGB-ALIKE, SuperPoint). Across both synthetic homography and real-pair pose evaluation, hyperspectral methods generally provide stronger geometric correspondences than RGB baselines in these texture-poor, specular conditions typical of surgical scenes.

On the ex vivo robotic dataset (Table 2), HS-ALIKE improves over RGB-ALIKE on planar localisation and matching, increasing Rep AUC (74.08 vs. 70.93) and MS AUC (67.21 vs. 64.05) while remaining competitive on MMA/MHA. Similarly, on real non-planar video pairs (Table 3), HS-ALIKE attains higher mAA@10$^\circ$ than RGB-ALIKE (63.14 vs. 61.56), indicating improved downstream pose recovery from the recovered correspondences. Although HSSPN does not consistently outperform its RGB analogue in every metric, it remains competitive (for example, mAA@10$^\circ$ 59.20 vs. SuperPoint 49.98), suggesting that even a 2D CNN trained directly on HSI can benefit from spectral cues.

These trends are consistent with the underlying characteristics of the modality. Compared to RGB, HSI provides additional wavelength information that can help differentiate between visually homogeneous tissue regions and reduce descriptor ambiguity when spatial texture is limited. As a result, hyperspectral keypoints and descriptors can remain more distinctive under challenging MIS imagery.

## Conclusion

We presented HyKey, a novel HSI-based framework for keypoint detection and description in MIS, utilising hybrid 3D-2D convolutions for joint spatial-spectral feature learning. Through geometry-aware training and benchmarking against RGB baselines, HyKey demonstrates that spectral information enhances feature localisation and matching robustness in challenging MIS scenes. Experiments on the proposed dual-modality dataset show consistent improvements in keypoint repeatability, matching precision, and pose recovery, highlighting the potential of HSI for reliable geometric perception in surgical vision. Future work will extend HyKey towards dense reconstruction pipelines using HSI-based SfM in real dynamic MIS scenes, as well as towards expanding to other HSI medical systems with different spectral ranges and laparoscopes.
